# Long-acting RNAi therapeutics in hypertension: limitations of single-target AGT silencing and rational multi-target synergistic strategies

**DOI:** 10.3389/fphar.2026.1943398

**Published:** 2026-09-17

**Authors:** Yi Lan, Xiaoxia Ye, Qingwei Zeng, Wenlong Zhou

**Affiliations:** 1 Department of Pharmaceutical and Medical Devices, Ganzhou Dermatology Hospital, Ganzhou, Jiangxi, China; 2 Department of Dermatology, Ganzhou Dermatology Hospital, Ganzhou, Jiangxi, China; 3 Third Inspection Office, Jiangxi Drug Inspection Center, Nanchang, Jiangxi, China; 4 Department of Medical Affairs, Guangzhou Xinhui Biotechnology Co., Ltd., Guangzhou, Guangdong, China

**Keywords:** angiotensinogen, GalNAc delivery, hypertension, multi-target oligonucleotide, reversibility, small interfering RNA

## Abstract

Hypertension affects over 1.3 billion people worldwide, fewer than 20% achieve adequate blood pressure (BP) control. Poor long-term BP management accelerates irreversible damage to target organs, highlighting the urgent need for novel therapeutic approaches. Small interfering RNAs (siRNAs) are synthetic oligonucleotides that harness endogenous RNA interference machinery to silence specific disease-related genes with high sequence specificity, an approach that has opened new therapeutic avenues in cardiovascular medicine by enabling durable target modulation with infrequent dosing. N-acetylgalactosamine (GalNAc)-conjugated long-acting siRNA agents address two major limitations of oral antihypertensives: poor patient daily adherence and short-lived BP reduction. However, nearly all clinical candidates are designed to silence hepatic angiotensinogen (AGT). Single-target inhibition cannot fully overcome the compensatory signaling networks that drive primary hypertension, limiting protection against progressive target-organ damage. Existing reviews have largely summarized AGT-targeted RNA interference (RNAi) therapeutics. Rather than cataloguing individual agents, we propose a phenotype-matched framework that uses disease mechanisms to guide target selection, delivery strategies, and translational development toward precision RNAi therapy for hypertension. We review current clinical progress, examine the mechanistic basis of multi-pathway blockade, and discuss emerging molecular scaffolds for dual- and multi-target siRNA therapeutics. Finally, we discuss the major barriers to clinical translation—including extrahepatic delivery, pharmacological reversibility, and safety considerations—and identify the advances required for phenotype-guided RNAi therapies to move toward precision cardiovascular medicine. We emphasize that this phenotype-matched model is intended to generate hypotheses for target prioritization and validation, and does not represent a clinically established treatment strategy.

## 1 Introduction

Hypertension affects more than 1.3 billion people globally and remains the leading reversible risk factor for severe cardiovascular events and chronic kidney disease (CKD) (NCD Risk Factor Collaboration, 2021; World Health Organization, 2023). Stable long-term BP management is essential to reduce hypertension related organ damage (HMOD) (World Health Organization, 2021). Despite this, conventional oral antihypertensives show limited real-world performance. Poor daily medication adherence and obvious circadian BP fluctuations independently worsen HMOD and raise risks of major adverse cardiovascular events (MACE) (Laurent, 2017; Burnier and Egan, 2019; Parati et al., 2013). Substantial proportions of hypertensive individuals remain undiagnosed even among attendees of primary care facilities (Evangelidis et al., 2025), highlighting the gap between clinical need and routine detection. Furthermore, many patients with resistant hypertension cannot reach standard BP goals despite optimized multi-drug regimens (Ettehad et al., 2016), creating high demand for novel ultra-long-acting antihypertensive treatments.

In cardiovascular disease, siRNA therapy provides a way to directly suppress selected pathogenic genes at the post-transcriptional level. Its prolonged gene-silencing effect also makes it well suited to conditions such as hypertension, in which continuous control of disease-driving pathways is often required (Ranasinghe et al., 2023). One subcutaneous injection can maintain therapeutic effects for months, removing reliance on daily patient adherence and alleviating the fluctuating BP seen with short-acting oral medicines (Cruz-López et al., 2022; Ye et al., 2023). Although these siRNA drugs have delivered promising clinical outcomes, nearly all candidates only inhibit hepatic AGT to block renin–angiotensin–aldosterone system (RAAS) signaling, which cannot offset the complex compensatory pathways of primary hypertension (Azizi et al., 2026; Díaz-Morales et al., 2023; Feng et al., 2025). At present, few systematic frameworks guide synergistic multi-target RNAi design. This review therefore dissects the limitations of single-target long-acting siRNA, summarizes design strategies and molecular structures of multi-target RNAi, and outlines major pharmacological obstacles hindering clinical transformation. Building on this analysis, we propose a phenotype-matched framework for multi-target RNAi design that is intended to generate testable hypotheses and guide future experimental validation.

## 2 The rise of long-acting RNAi: hepatic AGT silencing

Benefiting from the advantages of long-acting RNAi technology, AGT-targeted siRNAs have become the most advanced long-interval antihypertensive candidates. In the past decade, dozens of AGT siRNA candidates have entered clinical trials globally. Zilebesiran is the most mature candidate with abundant clinical data (Azizi et al., 2026), alongside multiple investigational agents under evaluation. Table 1 summarizes the global R&D pipeline of AGT siRNA therapeutics, and Supplementary Table S1 lists detailed clinical trial results (Bakris et al., 2024; Desai et al., 2025; SanegeneBio, 2025; Salubris Pharmaceuticals, 2026).

**TABLE 1:** Global R&D pipeline of AGT siRNA drugs for hypertension.

Company	Drug name	Phase	Target	Delivery platform	Trial ID	Registration date
Alnylam/Roche	Zilebesiran	Phase III	AGT	GalNAc delivery system	NCT07181109	18 September 2025
					NCT07553442	28 April 2026
					CTR20261053	20 March 2026
BeBetter Pharma	BEBT-701	Phase II	AGT/PCSK9	GalNAc delivery system	NCT07368608	26 January 2026
					CTR20260395	13 February 2026
GoWell Pharma/Salubris	GW906	Phase II	AGT	GalNAc delivery system	CTR20254537	13 November 2025
					CTR20262143	29 May 2026
Tianlong Pharma	YKYY029	Phase II	AGT	GalNAc delivery system	CTR20261322	9 April 2026
Leaderna Therapeutics	LDR2402	Phase II	AGT	GalNAc delivery system	CTR20253348	27 August 2025
Innovent/SanegeneBio	IBI3016	Phase II	AGT	GalNAc delivery system	NCT07352969	20 January 2026
					CTR20260014	15 January 2026
Hengrui Medicine	HRS-9563	Phase II	AGT	GalNAc delivery system	NCT07297797	22 December 2025
					CTR20254927	16 December 2025
Argo Biopharma/Novartis	BW-00163	Phase II	AGT	GalNAc delivery system	NCT07543120	21 April 2026
					CTR20233485	1 November 2023
SynerK/Hua DongMedicine	SNK-2726	Phase I	AGT	GalNAc delivery system	CTR20251753	30 April 2025
CSPC Pharma	SYH2062	Phase I	AGT	GalNAc delivery system	NCT06842537	24 February 2025
					CTR20250311	24 January 2025
Arnatar Biotech	ART101	Phase I	AGT	GalNAc delivery system	NCT07614308	29 May 2026
					CTR20253973	14 October 2025
Minwei/Lepu Medical	MWX401	Phase I	AGT	GalNAc delivery system	CTR20260870	6 March 2026
Sun-Novo/Anlong Bio	ABA001	Phase I	AGT	GalNAc delivery system	CTR20261621	29 April 2026
Rona Therapeutics	RN1871	IND	AGT	GalNAc delivery system	Not applicable	Not applicable
Sirnaomics	STP-237G	Preclinical	AGT/ApoC3	GalNAc delivery system	Not applicable	Not applicable
Corsera Health	COR-2003	Preclinical	AGT	Proprietary technology	Not applicable	Not applicable
CRISPR Therapeutics AG	CTX-340	Preclinical	AGT	Lipid nanoparticle	Not applicable	Not applicable
Sirnaomics	STP-136G	Preclinical	AGT	GalNAc delivery system	Not applicable	Not applicable
Suzhou Siran Biotech	SA-016	Preclinical	AGT	Not applicable	Not applicable	Not applicable

Clinical trial identifiers and registration dates were collected from ClinicalTrials.gov, the China National Clinical Trial Registry and Cortellis Drug Discovery Intelligence (data cutoff: July 2026). Abbreviations: AGT, angiotensinogen; ApoC3, apolipoprotein C3; GalNAc, N-acetylgalactosamine; IND, investigational new drug (phase); PCSK9, proprotein convertase subtilisin/kexin type 9.

As Supplementary Table S1 demonstrates, AGT siRNA maintains steady circadian BP and reduces morning BP spikes, overcoming the short trough efficacy of oral antihypertensives (Azizi et al., 2026; Desai et al., 2023). Collectively, these data confirm liver AGT silencing as a durable treatment for patients with low medication adherence and demonstrate the value of long-acting injectable antihypertensives. Table 1 also lists dual-target RNAi candidates for patients with hypertension combined with dyslipidemia. Even so, single-target hepatic AGT siRNAs still dominate clinical pipelines, and their antihypertensive effects cannot fully compensate for the drawbacks of single-pathway RAAS inhibition, which will be elaborated in the following section.

## 3 Ceiling effects and intrinsic limitations of single-target AGT RNAi therapy

While single-target AGT siRNA therapies have yielded encouraging clinical outcomes, multiple inherent pharmacological and technical bottlenecks are likely to hinder their widespread clinical translation and long-term utility.

### 3.1 Mechanistic constraints: network crosstalk and multifactorial pathogenesis

Early studies regarded RAAS as a linear pathway driving high BP (Vogt et al., 2025). In contrast, the updated mosaic theory of hypertension (visualized in Figure 1; Supplementary Figure S1) frames hypertension as a multifactorial network disease caused by coordinated dysfunction of renal, neural, vascular, metabolic, immune and genetic/epigenetic pathways (Harrison et al., 2021). Supplementary Table S2 outlines these interrelated pathological axes and corresponding molecular targets (Prieto et al., 2021; Hoorn et al., 2020; Verma and Feng Earley, 2025; DiBona and Kopp, 1997; Ja et al., 2023; Paul et al., 2006; Hall et al., 2019; Scheja and Heeren, 2019; Guzik et al., 2024; Niu et al., 2026; Zhang and Sun, 2021; Cheng and Zhang, 2010; Stölting et al., 2026). Interactions among these pathways initiate compensatory feedback mechanisms that contribute to the persistent elevation of BP and the progression of organ injury (Harrison et al., 2021). Thus, liver-only AGT inhibition cannot cover all heterogeneous hypertension subtypes, leaving many patients with uncontrolled BP and progressive organ damage.

**FIGURE 1:**
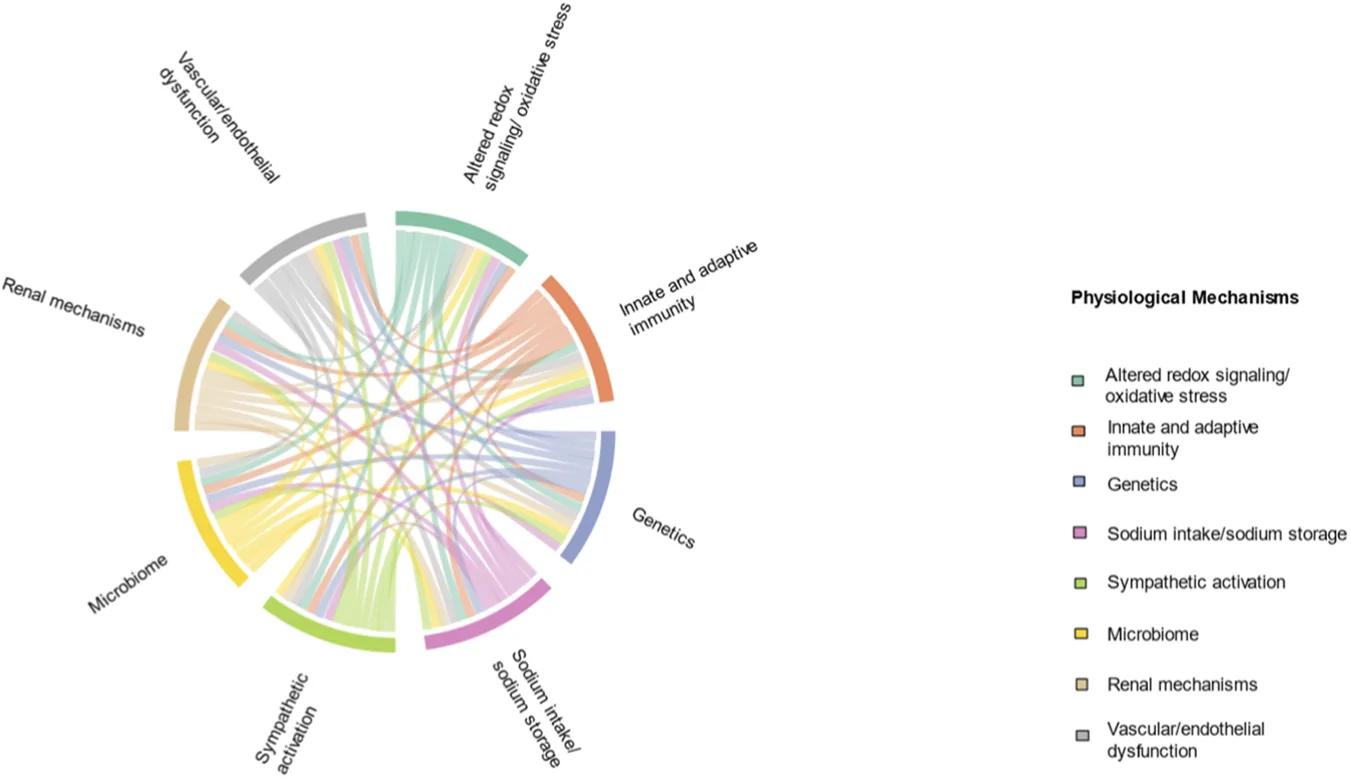
Mosaic theory of hypertension. Chord plot showing the bidirectional interactions among eight major pathophysiological axes involved in hypertension. Colored segments denote individual mechanistic modules, and links of equal width indicate qualitative reciprocal interactions without quantitative weighting. The chord plot was generated using the R package circlize.

### 3.2 Clinical heterogeneity: therapeutic challenges in volume-dependent hypertensive phenotypes

Phase II KARDIA trials confirmed that Zilebesiran could suppress hepatic AGT by over 90% across all doses, yet its antihypertensive efficacy varied greatly among patient subgroups. This variability was particularly evident in the KARDIA-3 trial, which enrolled high-risk patients with uncontrolled hypertension and comorbid conditions, such as chronic kidney disease. At month 3, the trial reported office SBP reductions of −5.0 mmHg (300 mg) and −3.9 mmHg (600 mg) relative to placebo. However, the study did not meet its pre-specified statistical significance threshold, as the hierarchical testing procedure required both dose groups to achieve p < 0.05. These modest and statistically inconclusive results highlight the therapeutic challenges posed by volume-dependent and comorbid phenotypes, where single-agent RAAS suppression may be insufficient to overcome compensatory mechanisms—such as altered renal sodium handling or heightened sympathetic drive—that sustain blood pressure elevation. The KARDIA-2 data further support this notion by demonstrating that Zilebesiran achieved optimal blood pressure reduction only when combined with a diuretic, suggesting that concurrent modulation of renal sodium transport is necessary for adequate efficacy in resistant hypertensive populations (Azizi et al., 2026; Bakris et al., 2024; Desai et al., 2025). Current hypertension guidelines recommend multi-drug combinations with complementary mechanisms for resistant cases, including triple therapy of CCB + ARB + diuretic, with diuretics serving as core combination partners in standard antihypertensive regimens (Supplementary Table S3) (Mancia et al., 2023; Writing et al., 2025). Beyond these efficacy-related considerations, the interpretation of the current evidence base is further constrained by the modest sample sizes and short follow-up durations typical of phase II trials, which preclude definitive assessment of long-term cardiovascular outcomes or mortality benefits. These inherent uncertainties underscore the rationale for ongoing large-scale phase III and IV studies designed to evaluate the long-term efficacy and safety of Zilebesiran across broader and more diverse patient populations.

### 3.3 Comorbidity-level limitations: unmet therapeutic needs in cardio-renal comorbidities

Hypertension frequently coexists with cardiovascular, renal, and metabolic comorbidities, including CKD, HF, T2DM, and dyslipidemia (Supplementary Figure S1). Such multimorbidity constitutes the primary driver of long-term MACE across hypertensive populations (World Health Organization, 2021; Writing et al., 2025). Early phase II KARDIA-1 and KARDIA-2 trials systematically excluded patients with severe renal insufficiency, decompensated HF, and poorly regulated metabolic disorders; moreover, although KARDIA-3 recruited participants with mild-to-moderate CKD and established cardiovascular disease, individuals with end-stage organ dysfunction remained ineligible across all phase II cohorts (Azizi et al., 2026; Bakris et al., 2024; Desai et al., 2025). This restrictive trial eligibility, coupled with the single-target RAAS-inhibitory mechanism of Zilebesiran, creates significant unresolved therapeutic gaps for patients with complex cardio-renal-metabolic phenotypes requiring multi-organ protective therapy. Hepatic AGT silencing effectively lowers systemic BP in populations with few comorbidities (Morosan et al., 2025). However, in hypertensive patients with coexisting cardiovascular and renal comorbidities, evidence remains insufficient to show that this strategy can simultaneously prevent progressive target-organ injury and improve accompanying metabolic abnormalities (Azizi et al., 2026).

### 3.4 Limitations of GalNAc-conjugated therapeutics in tissue targeting

The tissue selectivity of currently available delivery carriers remains a major constraint on the clinical application of RNAi drugs (Paunovska et al., 2022) Most RNAi therapeutics currently in clinical development employ GalNAc conjugation (Table 1) for hepatocyte-specific delivery via ASGPR. A representative example is Zilebesiran, whose hepatic targeting mechanism is shown in Figure 2 (Azizi et al., 2026). However, functional ASGPR is predominantly expressed in hepatocytes, limiting the use of this platform for gene silencing in extrahepatic cardiovascular and renal tissues (Roberts et al., 2020). A recent review published in Hypertension (2026) highlighted that AGT is also expressed in extrahepatic tissues, including renal tubules, adipose tissue, astrocytes, and the vascular wall, where it contributes to distinct forms of hypertension. These findings point to the need for RNAi delivery strategies that can effectively target non-hepatic tissues in cardiovascular disease (Kanbay et al., 2026).

**FIGURE 2:**
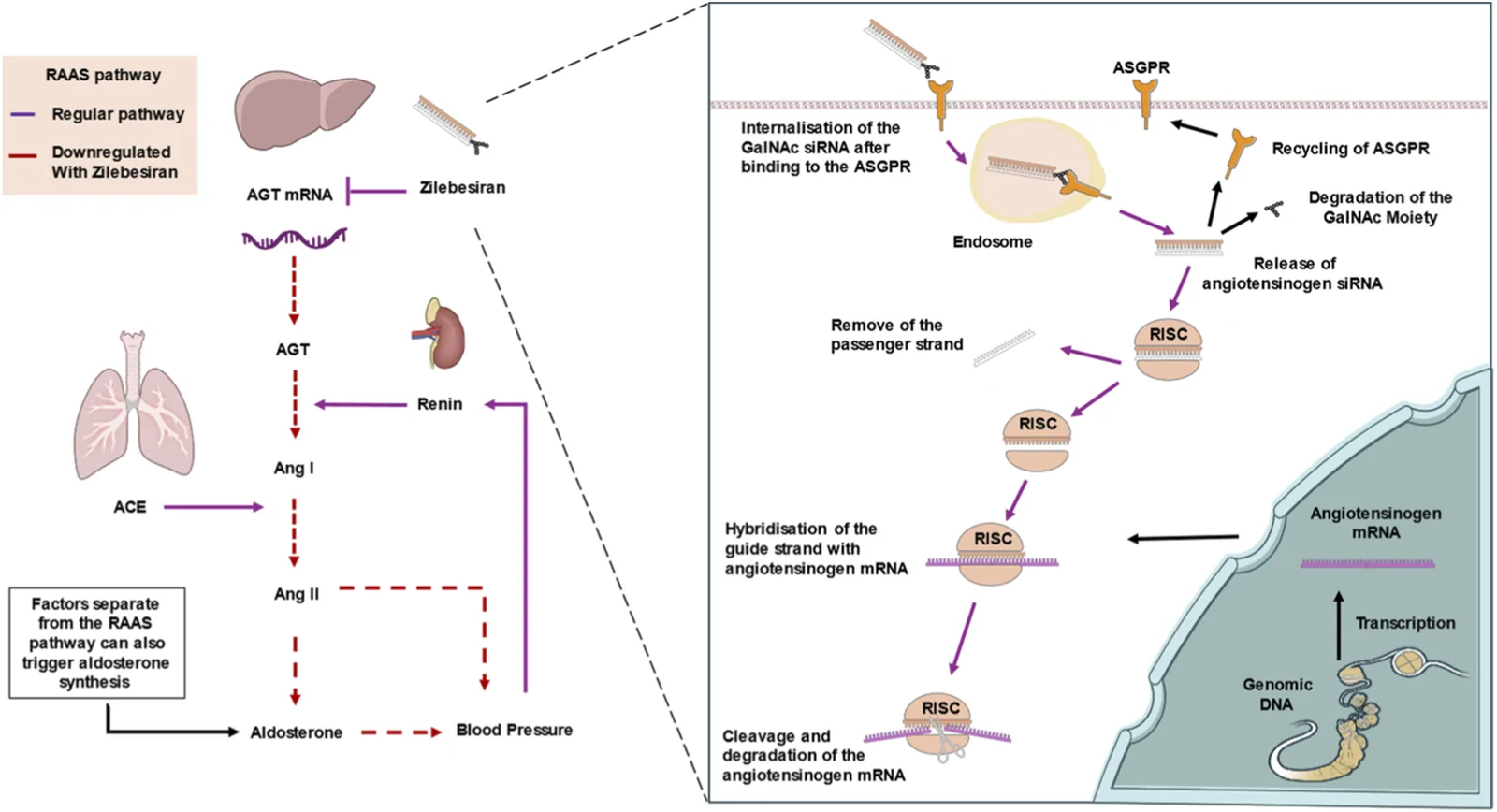
Mechanism of action of Zilebesiran. (Left) The RAAS pathway and hepatic AGT mRNA silencing induced by Zilebesiran. Purple solid lines indicate intact physiological signaling, whereas red dashed lines indicate suppressed signaling pathways. (Right) Delivery process of GalNAc-conjugated siRNA: (1) ASGPR-mediated cellular uptake; (2) endocytosis followed by endosomal escape; (3) loading of the antisense strand into the RISC complex; and (4) Ago2-mediated cleavage of AGT mRNA. Abbreviations: ASGPR, asialoglycoprotein receptor; GalNAc, N-acetylgalactosamine; RISC, RNA-induced silencing complex; Ago2, Argonaute-2; AGT, angiotensinogen; RAAS, renin–angiotensin–aldosterone system; siRNA, small interfering ribonucleic acid; mRNA, messenger ribonucleic acid.

Overall, the clinical translation of single-target AGT siRNA is limited by four major challenges: complex pathogenic networks, interindividual variability in therapeutic response, cardiometabolic comorbidities, and the liver-restricted tropism of GalNAc carriers. Neither dose optimization nor combination with conventional oral medications adequately addresses these limitations. These challenges provide a mechanistic rationale for the development of synergistic multi-target RNAi strategies, which are discussed in the following section.

## 4 A network pharmacology-guided framework for phenotype-matched multi-target RNAi design

The clinical success of oral antihypertensive therapies that combine multiple mechanisms of action provides a useful translational framework. By targeting complementary pathways, these combinations reduce compensatory responses and improve therapeutic efficacy across diverse hypertensive populations (King et al., 2026; Gong et al., 2026). Rather than adopting universal dual-target regimens for all patients, we favor individualized multi-target RNAi strategy selection according to predominant pathogenic mechanisms and unique clinical phenotypes of hypertensive individuals. By integrating the mosaic theory of hypertension, patient heterogeneity, contemporary translational evidence, and tissue delivery accessibility, we construct a network pharmacology-based platform for phenotype-adaptive multi-target RNAi design. This analytic system connects mechanistic disease signatures with rational target prioritization, targeted delivery optimization, and translational implementation, providing a rigorous theoretical basis for advancing precision RNAi therapeutics in hypertension.

The standardized workflow follows a five-step, phenotype-adaptive pipeline (Figure 3), progressing from clinical phenotyping and pathway identification to synergistic target screening, delivery system customization, and comprehensive translational assessment.

**FIGURE 3:**
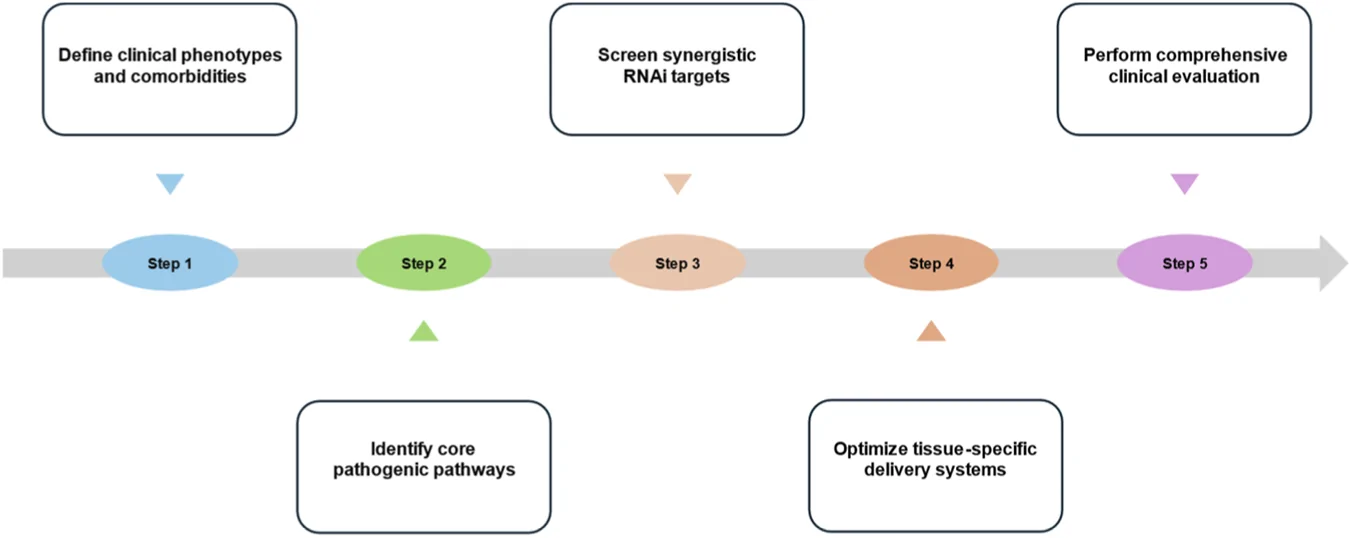
Workflow of the phenotype-matched multi-target RNAi design framework for hypertension. Step 1. Define dominant clinical phenotypes and concomitant comorbidities in individual hypertensive patients; Step 2. Identify core pathogenic pathways driving hypertensive progression and pathological deterioration; Step 3. Screen synergistic RNAi targets to concurrently modulate multiple interconnected pathological axes; Step 4. Optimize tissue-specific delivery systems tailored to the anatomical distribution of target organs; Step 5. Comprehensive assessment of the translational feasibility, biosafety, therapeutic reversibility, and clinical applicability of constructed RNAi regimens.

The following examples are therefore presented as evidence-graded therapeutic hypotheses rather than established treatment strategies.

### 4.1 Representative applications of the phenotype-matched platform

Several target combinations can be rationalized from the pathogenic mechanisms and clinical phenotypes of hypertension. However, the supporting evidence for these combinations is heterogeneous—ranging from active clinical development to preclinical validation or indirect mechanistic inference. Rather than providing an exhaustive classification, we use the following examples to illustrate how the phenotype-matched framework can guide rational target selection and combinatorial strategy design.

#### 4.1.1 Volume–resistance axis combination (AGT + ENaC/NCC) for salt-sensitive resistant hypertension

In salt-sensitive, volume-dependent hypertension, increased renal sodium reabsorption contributes substantially to persistent BP elevation (Prieto et al., 2021; Ramkumar et al., 2014). While hepatic AGT silencing with Zilebesiran broadly suppresses systemic RAAS signaling, high-salt dietary challenge data suggest salt-sensitive sodium-retention pathways persist and can blunt its antihypertensive efficacy (Desai et al., 2023). Concurrent modulation of renal sodium transport pathways, particularly ENaC or NCC, is therefore a mechanistically attractive way to complement RAAS suppression (Supplementary Table S2). Clinical data from KARDIA-2 provide indirect support for combining AGT suppression with diuretic-mediated sodium handling, but they do not establish renal ENaC/NCC co-silencing as an RNAi strategy (Desai et al., 2025). Even so, unlike hepatotropic GalNAc carriers used for hepatic AGT silencing, clinical transformation of this regimen depends on novel renal-targeted delivery systems to achieve kidney-specific ENaC/NCC silencing, which remains an unmet technical hurdle.

#### 4.1.2 Hepatic dual-gene silencing (AGT + PCSK9/ApoC3) for hypertension with dyslipidemia

Hypertension frequently coexists with dyslipidemia, accelerating atherosclerotic cardiovascular disease (Lauder et al., 2023; Watson and Fonarow, 2026). ApoC3 and PCSK9 regulate triglyceride-rich lipoproteins and atherogenic lipoproteins, whereas AGT serves as the rate-limiting substrate of the RAAS, supporting their selection as therapeutic targets. Because all three are predominantly synthesized in hepatocytes, they can be co-silenced through a shared GalNAc-based delivery platform. This strategy enables simultaneous reduction of BP and atherogenic lipids (Biessen and Van Berkel, 2021). Consistent with this advantage, BEBT-701 (AGT/PCSK9 dual-target siRNA, Phase II) and preclinical STP-237G (AGT/ApoC3 dual-target siRNA) have entered clinical and preclinical development (Table 1). Among these, the AGT + PCSK9 dual-target approach is currently one of the most clinically advanced multi-target RNAi candidates for hypertension with dyslipidemia. However, its Phase II status reflects safety and dosing assessments rather than proof of antihypertensive or lipid-lowering efficacy; no clinical outcome data are publicly available at this time.

#### 4.1.3 Metabolic axis-based combination (AGT + SGLT2/GLP-1 pathway) for T2DM and obesity

SGLT2 inhibitors and GLP-1 receptor agonists (GLP-1RAs) exert antihypertensive effects that are independent of their glucose-lowering actions. SGLT2 inhibitors rapidly lower SBP (2.5–4.0 mmHg) and DBP (1.5–2.0 mmHg) through osmotic natriuresis, plasma volume contraction, and sustained reduction in total body sodium and intracellular calcium. In contrast, GLP-1RAs produce a more gradual hypotensive effect, mainly lowering SBP (1.8–5.1 mmHg) through weight loss, vasodilation, and central sympathetic inhibition. Both classes are indicated for hypertensive patients with T2DM and obesity, and, with conventional antihypertensives, provide additive BP control and synergistic cardioprotection (Siddiqi et al., 2025; Lee et al., 2020). These observations provide a mechanistic rationale for combining AGT-silencing RNAi therapeutics with SGLT2 inhibitors or GLP-1RAs in patients with concomitant metabolic disorders. However, this rationale currently rests on extrapolation from small-molecule drug data; direct evidence for such combination strategies involving RNAi agents has not yet been reported.

#### 4.1.4 Inflammatory axis combination (AGT + IL-6/TNF-α) for obesity-associated hypertension

Studies have demonstrated that AGT-derived peptides from adipose tissue regulate adipocyte differentiation and contribute to the development of obesity (Karlsson et al., 1998). Obesity initiates persistent systemic inflammation and excessive sympathetic discharge, which synergistically upregulate RAAS activity (Satou et al., 2018; Ozbek et al., 2024; Drummond et al., 2019). Additionally, obesity and high-fat diets act as pivotal metabolic stressors that induce astrocyte reactivity and central neuroinflammation. Persistent interactions among central neuroinflammation, sympathetic overactivity, and systemic low-grade inflammation drive the pathological processes underlying hypertension-associated cardiorenal injury (Stern et al., 2016). Ang II acts on astrocytic AT_1_ receptors in the paraventricular nucleus (PVN), inducing glial oxidative stress and enhancing sympathetic excitation within the PVN. The resulting increase in sympathetic outflow promotes neurohumoral overactivation in hypertension and HF, leading to progressive cardiac and renal target-organ injury (Stern et al., 2016; Yu et al., 2013). At present, however, this combination remains a mechanistic hypothesis. Its feasibility depends on the development of delivery systems capable of selectively targeting the relevant extrahepatic tissues and immune compartments, followed by direct experimental validation.

The representative combinatorial regimens outlined earlier illustrate the translational implementation of our phenotype-matched platform for actionable RNAi design. Table 2 further quantifies their existing evidence grades and translational readiness.

**TABLE 2:** Preclinical and clinical evidence hierarchy and translational maturity of multi-target RNAi therapeutics for hypertension.

Classification	Target	Preclinical evidence	Clinical evidence	Potential
Hypertension combined with dyslipidemia	AGT + PCSK9	BEBT-701: Humanized double-transgenic mice and cynomolgus monkey studies demonstrate robust pharmacodynamic activity, with simultaneous lowering of BP and LDL-C alongside complete toxicological profiling, as described in the BeBetter Med 2025 Annual Report (BeBetter Med Inc, 2026)	Phase II clinical trial is ongoing, no published clinical outcomes yet.	High
	AGT + ApoC3	STP-237G: Efficacy studies with cell culture and animal models have been completed, as reported in the Sirnaomics 2024 Annual Report (Sirnaomics Ltd, 2025)	No clinical trials have been initiated	Moderate
Salt-sensitive resistant hypertension	AGT + NCC	Lack direct evidence Correlative data: Angiotensin II upregulates NCC via the AT1R–WNK4–SPAK signaling axis (Castañeda-Bueno et al., 2012; San-Cristobal et al., 2009)	The co-administration of thiazide diuretics (NCC inhibitors) and Zilebesiran produced maximal synergistic reduction in BP (−12.1 mmHg) (Desai et al., 2025)	Low
	AGT + ENaC	Lack direct evidence Correlative data: AGT siRNA downregulated renal γ-ENaC, implicating a mechanistic link to ENaC-mediated sodium transport (Uijl et al., 2019); In spontaneously hypertensive rats, a high-salt diet reversed the BP-lowering effect of AGT siRNA (Uijl et al., 2022)	Lack direct evidence Correlative data: In the Phase 1 clinical trial of Zilebesiran, a high-salt diet reversed the BP-lowering effect of AGT siRNA, underscoring the importance of volume control mechanisms (Desai et al., 2023)	Low
Hypertensive patients with T2DM and obesity	AGT + SGLT2i, GLP-1 RA	Lack direct evidence Correlative data: OLETF rats exhibit local RAS activation that drives oxidative stress and progressive renal damage, whereas treatment with dapagliflozin remarkably lowered the urinary Ang II and AGT levels (Shin et al., 2016; Puglisi et al., 2021) Chronic exendin-4 treatment in rats reduces renal cortical Ang II and urinary AGT excretion and urinary excretion of AGT, while blockade of GLP-1 receptor with exendin-9 exerted opposite effects (Martins et al., 2020)	Lack direct evidence Correlative data: In human studies, GLP-1 infusion reduced Ang II by 19% (P = 0.003) (Skov et al., 2013). RAASi monotherapy combined with SGLT2i (HR = 0.599, 95%CI 0.583–0.615) or GLP-1 RA (HR = 0.513, 95%CI 0.490–0.536) lowered mortality risk. Sequential triple therapy (RAASi + SGLT2i followed by GLP-1 RA) conferred the largest reduction in all-cause mortality and fewer MAKE (HR = 0.257, 95%CI 0.233–0.284) (Casper et al., 2025)	Low
Obesity-associated hypertensive inflammation	AGT + IL-6, TNF-α	Lack direct evidence Correlative data: Ang II infusion induced milder hypertension in IL-6 knockout mice, verifying that IL-6 is essential for AGT/Ang II-mediated hypertension (Brands et al., 2010); TNF-α upregulates AGT via RelA activation (Brasier et al., 1996)	Lack direct evidence Correlative data: In the CANTOS trial, Canakinumab reduces cardiovascular events in atherosclerotic patients on RAASi-containing standard treatment, with no significant impact on BP (Rothman et al., 2020)	Low

Lack of direct evidence denotes the absence of interventional validation for the proposed combination strategy, including RNAi-based co-targeting or mechanistically matched therapeutic approaches. Correlative data refer to *in vitro* studies, observational findings, or indirect animal evidence without direct validation of the combined intervention. Translational maturity was qualitatively classified according to the overall supporting evidence: High, supported by advanced preclinical validation combined with Phase II, or later clinical development; Moderate, supported by preclinical efficacy studies and/or indirect clinical evidence but lacking direct validation of the proposed combination strategy; Low, supported mainly by mechanistic rationale with limited experimental validation; such combinations are presented as hypothesis-generating directions for future research rather than clinically established strategies. Abbreviations: T2DM, type 2 diabetes mellitus; BP, blood pressure; MAKE, major adverse kidney events; HR, and 95% CI, values were derived from real-world cohort studies.

### 4.2 Emerging molecular architectures for multi-target RNAi therapeutics

Although the mixture-based approach allows flexible dose adjustment, inconsistent tissue distribution and cellular uptake of co-administered siRNA strands lead to unstable gene silencing (Chen et al., 2026), a critical drawback of simple siRNA mixtures that may greatly limit their application in cardiovascular pharmacotherapy. Current studies have identified three classes of integrated single-molecule siRNA platforms, all of which provide greater pharmacological stability than conventional siRNA cocktails. The pharmacological characteristics of these three mainstream unimolecular multi-target siRNA modalities are summarized in Supplementary Table S4 (Alterman et al., 2019; Anand et al., 2025; Brown et al., 2019; Egli and Manoharan, 2023; Jang et al., 2022; Tang and Khvorova, 2024; Kim et al., 2005; Liu et al., 2008). These unimolecular multi-target siRNA scaffolds enable synchronous co-silencing of multiple disease-relevant genes within the same target tissue, such as AGT combined with PCSK9 or ApoC3. Application of these multi-target scaffolds to multi-pathway hypertension treatment would significantly boost therapeutic outcomes. However, clinical research regarding their antihypertensive application remains insufficient to date, and key pharmacological uncertainties—including the potential for RISC competition among concurrently silenced genes and the limited validation of extrahepatic co-targeting strategies—remain largely uncharacterized in human trials.

### 4.3 Extrahepatic delivery: the critical barrier to clinical translation

The clinical translation of multi-target RNAi networks remains markedly impeded by insufficient systemic oligonucleotide distribution to extrahepatic tissues. Conventional GalNAc delivery systems rely on ASGPR recognition to achieve exclusive hepatocyte tropism (Biessen and Van Berkel, 2021), they barely penetrate renal, vascular and cardiac tissues. This limitation leaves non-hepatic pathogenic drivers of hypertension persistently unsuppressed. Accordingly, the practical translational value of next-generation combinatorial co-targeting strategies relies not only on rational pathological matching of target genes but also on the tissue delivery efficiency of oligonucleotide carriers.

To expand tissue tropism beyond the liver, alternative modalities—including altered lipid nanoparticles (LNPs), antibody-RNA conjugates (ARCs), engineered extracellular vesicles (EVs), and functionalized Virus-Like Particles (VLPs)—are under active preclinical investigation (Supplementary Table S5) (Chen et al., 2026; Wang et al., 2023; Cochran et al., 2024; Kong et al., 2025; Ma et al., 2026; Peralta-Cuevas et al., 2025; Bachmann et al., 2025; Kim et al., 2026). Despite encouraging proof-of-concept models, these approaches have not yet resolved efficient, reproducible, and scalable RNAi delivery to cardiorenal and other extrahepatic tissues. Therefore, the rational design of multi-target therapeutic regimens and the development of delivery platforms that safely overcome extrahepatic tissue barriers remain essential for network-based therapeutic intervention, and optimized delivery technologies may strongly shape translational outcomes.

## 5 Safety, reversibility, and antidote strategies for clinical translation

The prolonged pharmacological activity of long-acting siRNA therapeutics can improve medication adherence, but it also raises important concerns about reversibility during acute cardiovascular emergencies (Azizi et al., 2026; Ye et al., 2024). These safety concerns may become even greater with multi-target regimens. Simultaneous silencing of key hemodynamic regulators and renal sodium transport pathways may eliminate compensatory pressor mechanisms, thereby increasing the risk of catecholamine-resistant vasoplegia during severe hypovolemia or hemorrhage (Azizi et al., 2026).

Oligonucleotide-based antidotes, including REVERSIR technology, represent a promising approach to address this challenge. These antidotes consist of fully modified single-stranded oligonucleotides that bind to the Ago2-associated guide strand through complementary base pairing, forming stable heteroduplexes that prevent mRNA cleavage and facilitate intracellular degradation of the siRNA (Ye et al., 2024; Zlatev et al., 2018; Mateus et al., 2024). Despite their importance for the clinical translation of multi-target siRNA therapeutics, further development remains limited by poor cellular permeability and the risk of off-target interactions. In addition, evidence from large-animal studies is still needed to establish their efficacy and safety in emergency clinical settings (Ren and Danser, 2025; Addison et al., 2023; Cruz-López et al., 2026).

A three-tiered clinical framework is outlined in Supplementary Table S6 to support standardized risk management by integrating pre-treatment risk stratification, dynamic monitoring, and targeted emergency interventions. Future studies should also focus on the development of dual-mode siRNAs with switchable silencing activity and tissue-specific antidotes to improve pharmacological control. In addition, harmonized regulatory standards and standardized emergency protocols will be essential for the safe clinical implementation of long-acting RNAi therapeutics.

In addition to hemodynamic monitoring, regulatory evaluation should consider the long-term consequences of sustained, potent gene silencing, including irreversible epigenetic remodeling of sodium homeostasis and disruption of endogenous compensatory feedback mechanisms. Future clinical trial protocols should also include functional stress testing to evaluate renin- and catecholamine-mediated emergency reserve and confirm that compensatory responses remain intact in patients receiving multi-target siRNA during acute challenges, such as hemorrhage or excessive sodium intake.

## 6 Future perspectives and conclusion

Encouragingly, combinatorial regimens that silence AGT and PCSK9 simultaneously open promising therapeutic avenues for the clinical management of complex hypertension with cardiometabolic comorbidities, offering a viable multi-target intervention strategy for hypertensive multimorbidity. Multi-target siRNA therapeutics represent a rational advance over conventional single-gene RNAi modalities, capable of overcoming the limitations of single-target silencing to address the pathogenic mechanisms underlying essential hypertension. While liver-targeted AGT siRNA has demonstrated robust clinical efficacy in sustaining long-term BP control, its therapeutic performance across heterogeneous and resistant hypertensive phenotypes still requires further clinical verification.

The clinical translation of these multi-target integrated siRNA platforms is hindered by the hepatotropic nature of mainstream delivery systems. Advancing precision cardiovascular RNAi relies on novel carriers that specifically target extrahepatic tissues, including renal tubules, vascular walls, and the central nervous system. Additionally, substantial progress in addressing tissue distribution and antidote-dependent pharmacological controllability in large-animal models is an essential prerequisite for subsequent clinical translation. Moreover, the clinical translation of multi-target RNAi therapeutics faces health-economic and competitive barriers. Manufacturing complexity—especially for unimolecular scaffolds and extrahepatic carriers—raises costs exceeding generic oral drugs, limiting access in low-resource regions. Cost-effectiveness models incorporating adherence gains, reduced hospitalizations, and polypharmacy avoidance are needed to define adoption thresholds, with initial targeting of patients uncontrolled on optimized triple therapy.

Furthermore, the coordinated development of regulatory strategies is critical to the success of multi-target RNAi platforms. Given the long-acting and irreversible nature of such therapies, regulatory authorities are expected to impose increasingly stringent requirements on switchable silencing activity in clinical trials. Therefore, we believe that drug development for these platforms would benefit from early, proactive collaboration with regulatory bodies to establish standardized evaluation criteria for long-term reversibility and compensatory reserve functions, thereby optimizing clinical trial design and mitigating translational risks. Beyond regulatory and economic considerations, the therapeutic positioning of multi-target RNAi must also be defined in the context of emerging competing modalities. Compared with renal denervation and other interventional approaches, RNAi offers target specificity and programmability but faces endosomal entrapment, immunogenicity, and extrahepatic delivery hurdles. We propose a complementary role within the therapeutic arsenal, pending comparative effectiveness trials using hard cardiovascular and renal outcomes.

Overall, this phenotype-matched framework provides a practical strategy for designing multi-target RNAi therapies according to disease mechanisms, patient characteristics, and tissue accessibility. By linking these elements with delivery technologies and clinical translation, it offers a rational direction for the future development of precision RNAi therapeutics for hypertension.

The proposed phenotype-matched framework should be regarded as a hypothesis-generating model that requires prospective validation. Its clinical value will ultimately depend on advances in multi-target siRNA platforms together with evidence from ongoing preclinical and clinical studies. Rather than representing a definitive therapeutic paradigm, this framework provides a testable basis for guiding the rational development and clinical evaluation of next-generation RNAi therapeutics for hypertension.

Future antihypertensive RNAi therapeutics are expected to evolve from single-gene silencing toward programmable, precision, multi-target regulatory platforms integrating tissue-specific delivery, reversible pharmacology, and phenotype-guided intervention (Figure 4).

**FIGURE 4:**
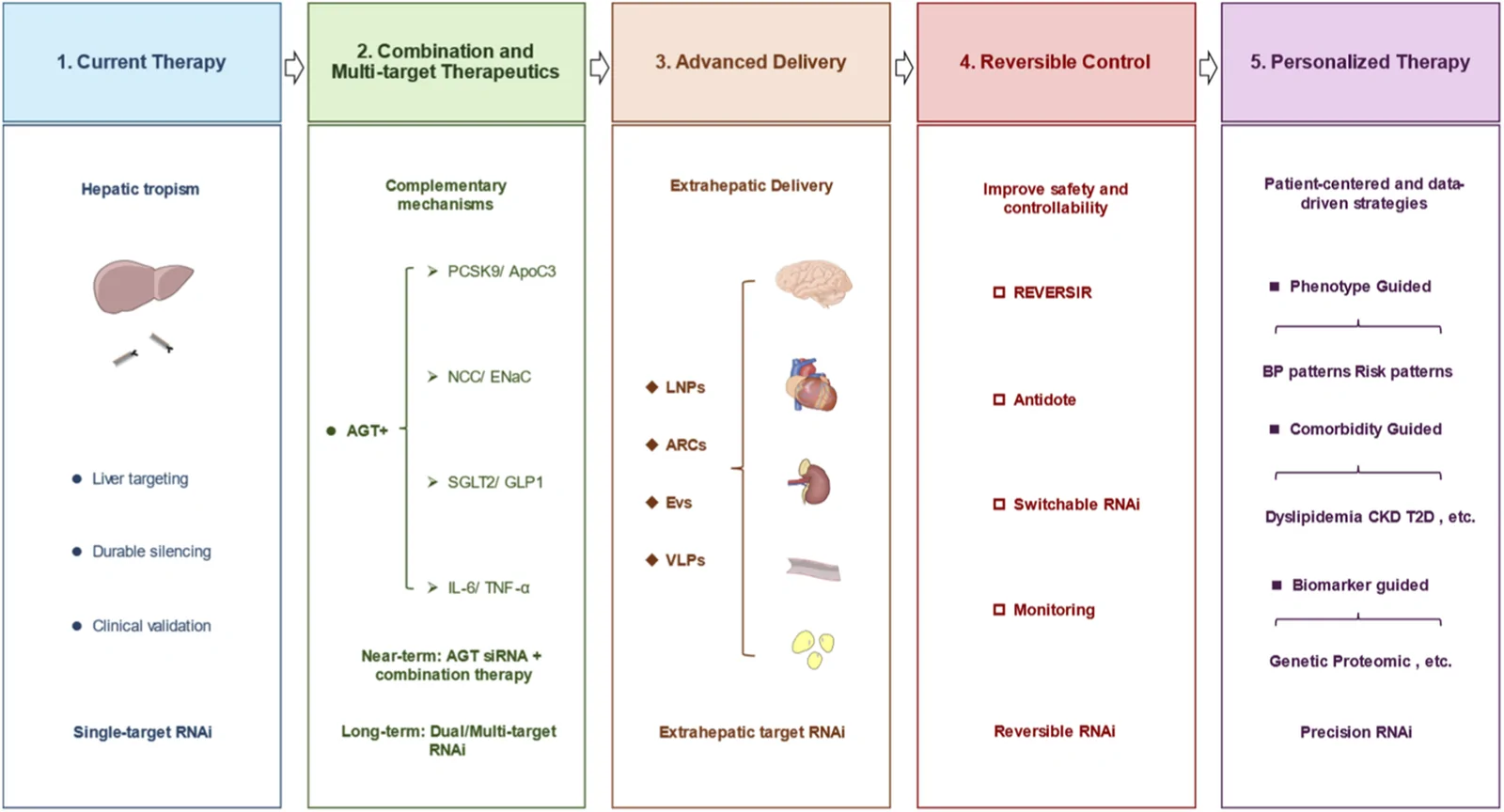
Conceptual framework for phenotype-guided multi-target RNAi strategies in hypertension. The roadmap tracks the shift from existing liver-targeted AGT siRNA treatments to multi-target and combination strategies, alongside advances in extrahepatic delivery, reversible RNAi, and personalized care. Early development primarily pairs AGT siRNA with conventional drugs, whereas future efforts will likely transition toward dual/multi-target platforms and precision medicine. Abbreviations: AGT, angiotensinogen; LNPs, lipid nanoparticles; ARCs, antibody-RNA conjugates; EVs, extracellular vesicles; VLPs, virus-like particles.

In summary, single-target AGT siRNA has proven capable of mitigating poor medication adherence through sustained long-term hypotensive effects, while multi-target network silencing remains an active area of preclinical and clinical research. Looking ahead, phenotype-guided multi-target RNAi strategies, supported by advances in delivery technologies and programmable RNAi platforms, may provide a practical foundation for precision antihypertensive therapy.

## Glossary

AGT: Angiotensinogen
ARB: Angiotensin II Receptor Blocker
ASGPR: Asialoglycoprotein Receptor
ApoC3: Apolipoprotein C3
ACE: Angiotensin-Converting Enzyme
ACEI: Angiotensin-Converting Enzyme Inhibitor
Ago2: Argonaute 2
**AT**_**1**_ **R**: Angiotensin II Type 1 Receptor
ARCs: Antibody-RNA Conjugates
BP: Blood Pressure
CKD: Chronic Kidney Disease
CCB: Calcium Channel Blocker
DBP: Diastolic Blood Pressure
ENaC: Epithelial Sodium Channel
EVs: Extracellular Vesicles
ET-1: Endothelin-1
FFA: Free Fatty Acid
GalNAc: N-acetylgalactosamine
GLP-1: Glucagon-Like Peptide-1
GLP-1RA: Glucagon-Like Peptide-1 Receptor Agonist
HMOD: Hypertension Related Organ Damage
HF: Heart Failure
IL-6: Interleukin-6
IL-17: Interleukin-17
IHD: Ischemic Heart Disease
LDL-C: Low-Density Lipoprotein Cholesterol
LNPs: Lipid Nanoparticles
LncRNAs: Long non-coding RNAs
MACE: Major Adverse Cardiovascular Events
mRNA: Messenger Ribonucleic Acid
MAKE: Major Adverse Kidney Events
NHE3: Sodium-Hydrogen Exchanger Isoform 3
NCC: Sodium-Chloride Cotransporter
NO: Nitric Oxide
PCSK9: Proprotein Convertase Subtilisin/Kexin Type 9
PVN: Paraventricular Nucleus
RNAi: RNA interference
RAAS: Renin-Angiotensin-Aldosterone System
R&D: Research and Development
RISC: RNA-induced silencing complex
siRNA: Small Interfering RNA
SGLT2: Sodium-Glucose Linked Transporter 2
SBP: Systolic Blood Pressure
SPAK: STE20/SPS1-Related Proline/Alanine-Rich Kinase
T2DM: Type 2 Diabetes Mellitus
TNF-α: Tumor Necrosis Factor-Alpha
TGF-β: Transforming Growth Factor-Beta
VLPs: Virus-Like Particles
WNK4: With No Lysine (K) Kinase 4
