# Supplementary material for: Long-acting RNAi therapeutics in hypertension: limitations of single-target AGT silencing and rational multi-target synergistic strategies
Source: Front Pharmacol. 2026 Sep 17;17:1943398. doi: 10.3389/fphar.2026.1943398 (PMC13626916; doi:10.3389/fphar.2026.1943398)
Supplement: Supplementary file 1 [file Supplementaryfile1.pdf]

Supplementary Materials

Supplementary Table S1. Clinical data summary of AGT siRNA pipeline drugs

Company	Drug	Knockdown (AGT) Data	Blood Pressure Reduction Data
Alnylam / Roche	Zilebesiran/ALN-AGT	KARDIA-2 Study (Phase II): After a single dose of 600 mg, serum AGT levels decreased by 87.8-92.8% at 2 weeks; the reduction was maintained at 88.2-94.5% at 6 months.	KARDIA-1 Study (Phase II Monotherapy): After 3 months of treatment, compared with placebo, 24-hour mean ambulatory SBP was reduced by 14.1-16.7 mmHg (across different dose cohorts);<br>KARDIA-2 Study (Phase II Combination Therapy): On top of existing standard-of-care treatments (e.g., Olmesartan), adding Zilebesiran additionally reduced SBP by 12.1 mmHg at Month 3 (compared with placebo) (Bakris et al., 2024; Desai et al., 2025).
Innovent / SanegeneBio	IBI3016/SGB-3908	Phase I Study: The maximum reduction (91.4-97.5%) was achieved approximately 4 weeks after a single dose. At 3 months, AGT levels across all dose cohorts continuously decreased by 90.0-97.0%; at 6 months, the reduction remained sustained at 84.0-96.4%.	Phase I Study: At 3 months, blood pressure reductions were observed in all dose cohorts. In the highest dose cohort, the 24-hour mean ambulatory SBP/DBP changes from baseline were -16.7/-14.7 mmHg (compared to -3.2/-5.7 mmHg in the placebo group), according to a SanegeneBio company press release (SanegeneBio, 2025).
GoWell Pharma/Salubris	GW906/SAL0132	Phase I Study (Released at ACC 2026): All dose cohorts were able to reduce AGT protein levels by more than 90%. In the lower dose cohort (Cohort 2), the effect of a single dose lowering AGT by >90% was sustained up to Month 9.	Phase I Study: In the potential therapeutic dose cohort (Cohort 3), a mean clinic BP reduction of approximately 25/13 mmHg (SBP/DBP) was achieved at Month 3. In another dose cohort (Cohort 4), a DBP reduction of 15.9 mmHg was observed at Week 2, and the reduction stably maintained up to Month 6, per a Salubris Pharmaceuticals company press release (Salubris Pharmaceuticals, 2026).
Other	therapeutic candidates	No public detailed data available.	No public detailed data available.

Supplementary Table S2. Core pathogenic pathways and associated therapeutic targets in hypertensive pathogenesis

Pathophysiological Mechanisms	Pathological Process	Key Clinical / Molecular Targets
Renal/Fluid Volume	Enhanced sodium and water reabsorption in renal proximal tubules impairs the pressure-natriuresis mechanism, directly driving salt-	Intrarenal AGT, NHE3, ENaC, NCC, and other ion channels/transporters (Prieto et

	sensitive hypertension.	al., 2021; Hoorn et al., 2020).
Sympathetic Nervous System	Elevated central sympathetic outflow stimulates intense peripheral vasoconstriction and triggers renal renin release.	Astrocytic AGT, α_1/β_1-adrenergic receptors, renal sympathetic nerve terminals (Verma and Feng Earley, 2025; DiBona and Kopp, 1997).
Vascular/Endothelial	Endothelial dysfunction leads to reduced NO bioavailability; mechanical stress and oxidative stress induce vascular smooth muscle cell proliferation and vascular remodeling.	Local vascular AGT, ACE, AT1-receptor, ET-1(Ja et al., 2023; Paul et al., 2006).
Metabolic/Humoral	Obesity and visceral adipocyte hypertrophy trigger paracrine release of excessive adipokines and local pressor factors, inducing sympathetic hyperactivation and insulin resistance.	Adipose tissue AGT, Leptin, FFA (Hall et al., 2019; Scheja and Heeren, 2019)
Immune/Inflammatory	Chronic immune activation leads to T-cell and macrophage infiltration into the kidneys and vascular walls, releasing pro-inflammatory cytokines that disrupt renal sodium excretion and vascular compliance.	IL-17, TNF-α, IL-6, TGF-β and their corresponding receptors (Guzik et al., 2024).
Genetic/Epigenetic	Polygenic susceptibility variants and environmental stimuli alter downstream target expression via DNA methylation, histone modifications, and non-coding RNAs.	M235T /-6G→A variants, miRNAs (e.g., miR-21), LncRNAs (Niu et al., 2026; Zhang and Sun, 2021; Cheng and Zhang, 2010; Stölting et al., 2026).

Supplementary Table S3. Molecular targets, pharmacological actions and guideline-endorsed combination regimens of classic antihypertensive drugs

Class	Mechanism	Target	Combination Strategy	Evidence Grade
ACEI	Inhibits ACE; reduces Ang II and bradykinin degradation	ACE	Combine with CCB or Thiazide-type diuretic	Class I, Level A
ARB	Blocks Ang II binding to AT1 receptor	AT1 Receptor	Combine with CCB or Thiazide-type diuretic	Class I, Level A
CCB	Blocks L-type calcium channels, induces vasodilation	L-type Ca^{2+} Channel	Combine with ACEI, ARB, or Thiazide-type diuretic	Class I, Level A
Diuretics	Inhibits Na^+/Cl^- cotransporter in distal tubule	Na^+/Cl^- Cotransporter	Combine with ACEI, ARB, or CCB	Class I, Level A
Beta-blockers	Reduces HR and contractility, inhibits renin release	β_1-Adrenoceptor	Reserved for specific indications (HF, IHD, Arrhythmia)	Specific Indications

Source: Based on 2023 ESH Guidelines and 2025 High Blood Pressure Guideline (Mancia et al., 2023; Writing et al., 2025).

Supplementary Table S4. Molecular architectures for multi-target RNAi therapeutics

Platform Type	Mechanism	Distinction

Branched / Multivalent siRNA Conjugates	Multiple siRNA cassettes linked to a common scaffold	Multi-siRNA tethered to non-cleavable permanent scaffold at synthesis; no intracellular cleavage (Alterman et al., 2019; Anand et al., 2025).
Chemically Linked Dual-siRNA Constructs	Covalently connected siRNAs released intracellularly	Dual siRNA coupled via reducible linkers; intracellular glutathione cleaves bonds to release free siRNAs (Brown et al., 2019; Egli and Manoharan, 2023).
Dicer-Substrate or Polycistronic RNAi Designs	Single precursor generates multiple active siRNAs	No pre-linked siRNA cassettes; single long precursor is enzymatically cut by intracellular Dicer to produce diverse siRNAs (Jang et al., 2022; Tang and Khvorova, 2024; Kim et al., 2005; Liu et al., 2008).

Supplementary Table S5. Summary of extrahepatic delivery platforms for RNAi therapeutics

Delivery Platform	Target Organs and Tissues	Mechanism	Challenges	Stage
Enhanced Lipid Nanoparticles (LNPs)	Vascular endothelium, Glomeruli	ligand-functionalization	Hepatic sequestration; potential immunogenicity	Preclinical / Early clinical (Chen et al., 2026; Wang et al., 2023)
Antibody-RNA Conjugates (ARCs)	Myocardium	Antigen-antibody specific binding	Complex synthesis; constrained conjugation efficiency	Clinical (Chen et al., 2026; Cochran et al., 2024)
Engineered Extracellular Vesicles (EVs)	Microglia	Membrane fusion; endogenous receptor-ligand interaction	Complex large-scale production; standardization issues	Exploratory research (Kong et al., 2025; Ma et al., 2026)
Functionalized Virus-Like Particles (VLPs)	Tumors, tumor-associated immune cells, vascular endothelial cells	Receptor-mediated endocytosis	Potential immunogenicity; scalability challenges	Preclinical (Peralta-Cuevas et al., 2025; Bachmann et al., 2025; Kim et al., 2026)

Supplementary Table S6. Graded management framework for siRNA-associated adverse reactions

Grade	Clinical Presentation	Recommended Strategy
I	Mild hyperkalemia, slight hypotension, local injection reaction	Symptomatic care
II	Dizziness, recurrent orthostatic hypotension	Volume regulation + adjuvant therapy
III	Refractory shock, organ damage, fetal risk	REVERSIR + comprehensive life support

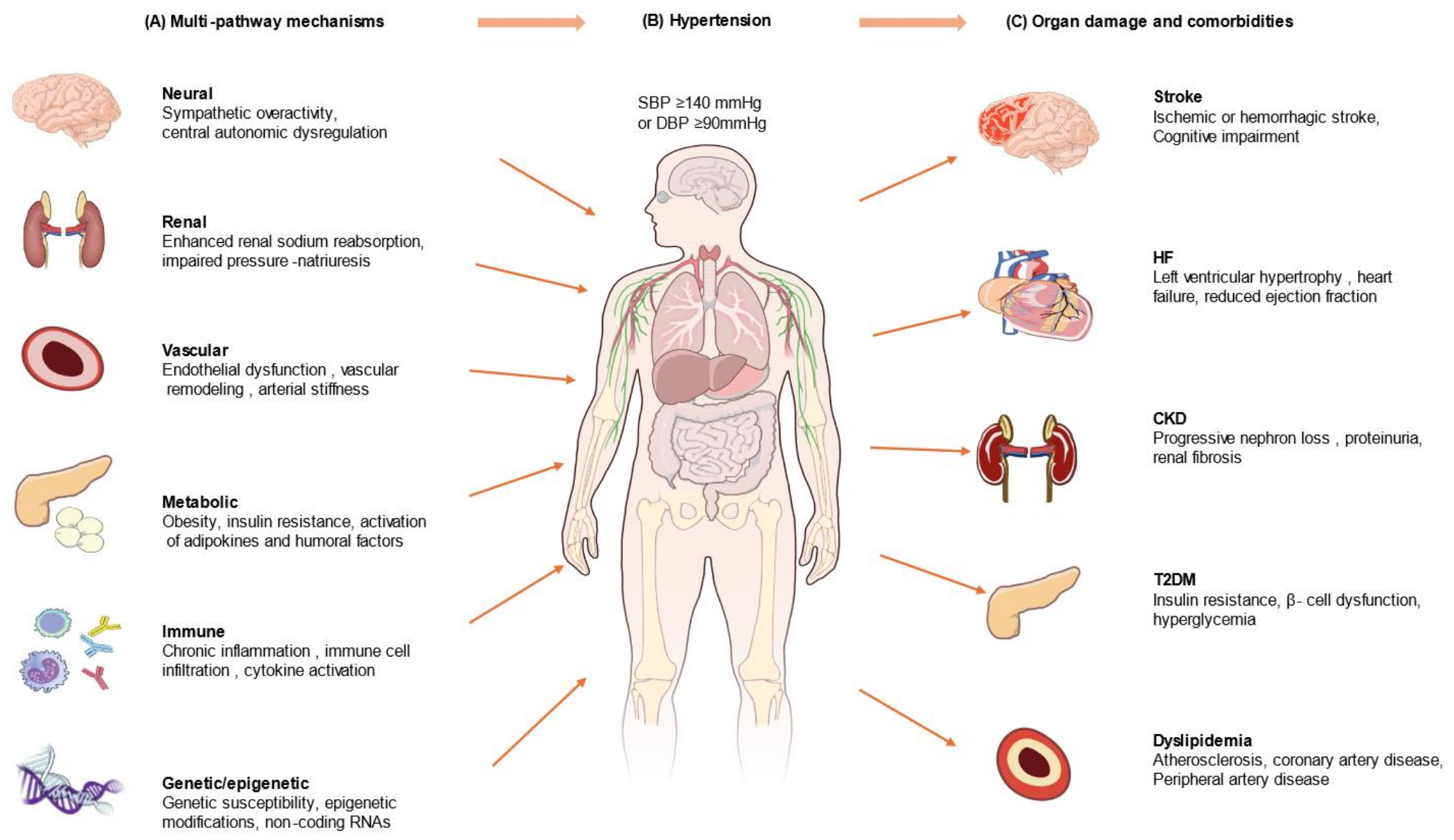

Supplementary Figure S1. Interconnected pathogenic networks driving hypertension and subsequent organ damage. Schematic representation of the multifaceted mosaic paradigm of essential hypertension, contrasting the conventional linear RAAS-centric model with an integrated multi-network pathogenic framework. Three distinct tiers are demarcated sequentially: (A) Diverse upstream physiological and molecular triggers contributing to BP elevation, encompassing neural, renal, vascular, metabolic, immune, and genetic/epigenetic pathways; (B) Sustained hypertension phenotype, SBP ⩾140 mmHg or DBP ⩾ 90mmHg; (C) Long-term hypertensive complications and cardio-metabolic comorbidities. All orange arrows uniformly denote causal promoting effects from upstream pathogenic drivers to systemic end-organ lesions. Abbreviations: RAAS, renin–angiotensin–aldosterone system; SBP, systolic blood pressure; DBP, diastolic blood pressure; HF, heart failure; CKD, chronic kidney disease; T2DM, type 2 diabetes mellitus.
